# Comparison of unidirectional barbed knotless suture versus vicryl rapide suture for intraoral wound closure in maxillofacial trauma: A clinical study

**DOI:** 10.6026/973206300221187

**Published:** 2026-02-28

**Authors:** Sarabpreet Kaur, Tejinder Kaur, Amit Dhawan, Sarika Kapila, Ramandeep Singh Bhullar

**Affiliations:** 1 Department of Oral and Maxillofacial surgery, Sri Guru Ram Das Institute of Dental Sciences and Research, Sri Amritsar, Amritsar, India

**Keywords:** Barbed suture, healing, maxillofacial trauma, suturing time, vicryl rapide

## Abstract

The challenge in maxillofacial trauma is achieving effective intraoral wound closure with optimal healing outcomes. Therefore, it is of interest to compare the efficacy of unidirectional knotless barbed suture with Vicryl Rapide suture for intraoral wound closure. Thirty fracture sites were divided into two groups, with group I using 3-0 knotless barbed suture and group II using 3-0 Vicryl Rapide. Results showed that suturing time was shorter in group I and wound healing were superior in group I at both 7 and 14 days. Thus, we show that knotless barbed suture is an effective choice for maxillofacial wound closure, requiring surgeons to understand suture biomechanics for optimal material selection.

## Background:

Wound care is of utmost importance in oral and maxillofacial trauma patients, as it plays a crucial role in the success of treatment. Effective wound closure materials are sought after to provide strength and stability, especially against the powerful muscles in the facial region [1]. Traditional sutures such as silk and Vicryl have been commonly used in maxillofacial surgeries. Silk, a non-absorbable suture material derived from silkworms, offers strength but requires removal after a certain period [2]. Vicryl, a synthetic absorbable suture made from polyglactin 910, has variants like Vicryl Plus, which includes added antimicrobial properties. These sutures dissolve over a period of 56 to 70 days, depending on the type. Among the Vicryl options, Vicryl Rapide/Velosorb fast is a multifilament suture with a faster absorption rate of 42 days. However, it is susceptible to bacterial colonization at knot sites, which can lead to infections [3]. In contrast, knotless sutures such as Stratafix and V-Loc have emerged as promising alternatives. These monofilament sutures feature barbs arranged in helical or spiral patterns along their entire length, which enhance tissue holding capacity and reduce the surface area available for bacterial growth [4]. Unlike traditional sutures, knotless barbed sutures eliminate the need for knots, simplify the closure process and reduce complications associated with knot tying. These sutures are available in both unidirectional and bidirectional types and have been increasingly used in various surgical procedures [5]. They have shown superior wound healing outcomes compared to traditional sutures, particularly in procedures like impacted mandibular third molar removal, head and neck reconstruction and MRONJ (Medication Related Osteoradionecrosis of the Jaw) [6 7-8]. As research on knotless barbed sutures continues to expand, they are poised to become integral in modern maxillofacial and dental surgeries. The potential for improved surgical outcomes, faster wound healing and a reduced risk of complications makes knotless sutures a valuable alternative to conventional suturing techniques [9]. Therefore, it is of interest to determine the effectiveness of knotless barbed sutures as a superior alternative to traditional sutures like Vicryl Rapide, offering potential benefits in wound healing, reduced complications and enhanced surgical outcomes in maxillofacial trauma patients.

## Materials and Methods:

The research encompassed 30 individuals who sought treatment for maxillofacial fractures at Oral and Maxillofacial Surgery Department. Patients were provided with a detailed explanation of the treatment plan and their participation in the study was contingent upon obtaining informed consent. Pertinent demographic information such as age, gender and relevant medical history of the patients were meticulously documented. The research protocol was approved by the Institutional Review Board and ethical committee. The study was conducted prospectively as a randomised controlled clinical trial. Healthy patients >16 years of age (ASA1 category) and patients who required intraoral open reduction and internal fixation for isolated maxillofacial fractures were included in the study. Patients who had prexisting medical conditions and on medicines that intervene the healing of the wound, patients who were allergic to any of the suture material, drugs or anesthetic agent used in the surgical protocol and patients with infected or avulsive wounds were excluded from the study The Sample was divided into two groups; Control (n=15) and Study (n=15) (Table 1). The patients were allocated to the Control and Experimental Groups through Random Numbers Generation Mechanism (via R-programming), as follows:

Firstly open reduction and internal fixation of all fractures was done using a standard surgical procedure in Oral and Maxillofacial Surgery Department of our, institute. Wound closure was achieved using unidirectional 3-0 Monocryl Knotless barbed suture (STRATAFIX Knotless Tissue Control Devices, ETHICON LLC, San Lorenzo Puerto Rico, United States) in the study group while 3-0 Vicryl Rapide suture (ETHICON NW2732, Johnson & Johnson private limited Aurangabad, India) was used in the control group. In group I, the mucoperiosteal flap was held and a deep bite in the muscle or periosteal layer from one side of the wound was taken. For securing the suture properly, the needle was moved through the preformed loop present at another end of the suture. A gentle traction was applied for properly approximating the wound edges. At last two back stitches were taken in the backward or reverse direction at a distance from the apex of the wound. Suturing technique in 3-0 vicryl rapide group varied from group I. In this suture there was no preformed loop, so knot was tied for securing the sutures.

## Intraoperative assessment:

Several parameters were evaluated in both groups, including the time taken for wound closure, which was recorded in minutes using a stopwatch to approximate the wound edges until the last suture. Other parameters assessed were breakage of the suture needle, suture breakage, needle stick injuries to the operator or assistant during suturing and the length of the suture material utilized, which was recorded in centimeters.

## Pain:

Pain was recorded on the 3rd, 7th, 14th and 21st days after surgery using the Numeric Rating Scale (NRS), where patients rated their pain from 0 to 10, with 0 indicating no pain and 10 representing the worst possible pain.

The NRS scores are;

Score 0 = no pain

Score 1-3 = mild pain

Score 4-6 = moderate pain

Score 7-10 = severe pain

## Wound evaluation:

Wound healing was evaluated on the 3rd, 7th, 14th and 21st days using the wound evaluation scale The wound was assessed based on six clinical variables, each with a score of 1/0 (Table 2).Each category was graded as zero or one on point scale. Overall score of 6 was considered as adequate healing, whereas score less than 5 or equal to 5 was considered as suboptimal closure of wound. All the measurements were done by a trained research coordinator 3 complications: Complications encountered during wound healing such as wound dehiscence and suture extrusion were documented. The collected data was subjected to the appropriate statistical analysis through customized programmes in R-language (Version 4.1.2) to determine the effectiveness of unidirectional 3-0 knotless barbed suture and 3-0 vicryl rapide suture, for closure of intraoral wound in trauma patients. Differences between mean values of wound closure time and suture length among the groups were evaluated using unpaired t-test. Difference between mean scores of pain was tested using Mann-Whitney u-test. Also difference in mean scores of wound healing at different points of time was tested using Mann-Whitney u-test. For all the statistical tests, p<0.05 was regarded as statistically significant.

## Results:

This study involved a total of 30 fracture sites in 73.3% males and 26.7% females with a mean age of 26.93+56.19 years in group I and 32.00+13.586 years in group II (Table 3). The time taken for suturing was considerably less in the study group than the control group. The study group demonstrated a mean (SD) suturing time of 3.809±0.253 minutes, compared to 6.923 ± 0.438mins in the control group suture length was 15.867 ± 0.617cm in Group I compared to 23.667 ± 1.253cm in Group II The difference in suturing time and length of suture material was highly significant between the study and control group (p value < 0.001) (Table 4). In addition to this the mean score of postoperative pain in group I and group II was noted at day 3rd, 7th, 14th and 21st. Pain was observed significantly less in group I as compared to group II at day 3 (p= 0.0144), day 7 (p= 0.0331) and day 14 (p= 0.0143). However there was no significant difference in between the two groups over mean pain score on post op day 21 (p =0.1090). Table 5 depicts comparison for studying association between the levels of wound healing in two groups at different points of time. We have first generated two way contingency tables (given below) on the frequencies of wound healing scores in both groups. On these tables Fisher's exact test was applied. The reason of applying this test was that the expected frequencies in some of cells of the contingency tables were lesser than 5. According to the test statistically significant difference was observed between both groups on post-operative day 7 (p = 0.00668) and day fourteen (p= 0.00778). Highly significant difference in wound healing was specified between two groups on 3rd post-operative day (p = 0.00014), However there was inconsiderable difference in wound healing on 21st postoperative day (p= 0.32950) between the groups (Table 6). Wound healing was observed to be better in the study group as per Mann Whitney U test results comparing the healing between the group I and group II on 3rd post-operative day (p =0.0932), 7th postoperative day (p=0.0018), 14th postoperative day (p=0.0248) and on 21st postoperative day (p= 0.1577). Difference in wound healing mean score between two groups was significant at both the 7th day and 14th day whereas it was marginally significant with 10% probability on day 3 and non-significant on day 21 (Table 7). Complications seen in the two groups were suture extrusion and wound dehiscence Suture extrusion was seen in 2 patients each (13.3%) in group I and group II whereas wound dehiscence was observed in 1 patient (6.7%) of group II (x2=1.040, df =2, p=0.595). No case of suture needle breakage or needle stick injury was seen in any of the groups. However, suture breakage was seen in 2 cases of group II (13.3%) (Table 8).

## Discussion:

In maxillofacial surgery, intraoral incisions are preferred to avoid extra oral scarring and nerve damage. Precise closure of these incisions is vital for successful outcomes. Throughout history, various materials like gold, silver, silk and synthetic biomaterials have been used for suturing [10]. Modern suturing materials aim to facilitate tissue apposition, support margins, maintain hemostasis, promote healing, prevent infection and ensure aesthetic results. Efficient closure techniques are essential for achieving these goals swiftly and effectively [11]. Conventional sutures have more surface area for bacteria to inhabit and leads to loss of tensile strength weakening the suture. These sutures necessitate knots to secure, to prevent them from loosening up and to maintain adequate tension across the wound margins. Knots leads to stretching and thinning of suture material. More tight knots may lead to necrosis thus affecting wound healing and impairs fibroblast proliferation knotted suture are associated with interuser variability. Being multifilament harbours more bacterias and causes irritation. To overcome these limitations and for better healing of the wound an alternative suture which does not require knots was developed and named as knotless barbed suture [12]. Alcamo was the first to submit the idea of barbed suture to the US patent office on August 13, 1956. Researchers initially documented the application of barbed sutures in human cadavers and animals. In 2004, FDA granted approval for the utilization of these sutures in diverse surgical procedures [13]. This innovative method of suturing is now being used in abdominoplasty procedures, facial rejuvenation, arthrotomy and laproscopic myomectomies etc., [14]. Moffa *et al.* (2023) [15] used the suture in pharnygoplasty and in case of obstructive sleep apnea patients. There are very less studies reported in literature regarding the intraoral use of knotless barbed suture. The study compared knotless sutures with Vicryl sutures in terms of time taken for suturing, suture length, postoperative pain and wound healing. The knotless suture group showed significantly shorter wound closure time compared to the Vicryl group. Similar results were found in studies by Ceyar *et al.* (2020) [16], which reported reduced suturing time with knotless sutures due to the presence of barbs and the absence of knots. Regarding pain, the knotless suture group had lower pain scores postoperatively, supporting the results of Migliorini *et al.* (2025) [17], where pain reduced significantly by day 7. Furthermore, wound healing was better in the knotless suture group on days 3, 7 and 14, as also noted in other studies. Lastly, while suture breakage was observed in the Vicryl group, studies, including Tozsin *et al.* (2023) [18], found barbed sutures to have superior tensile strength, with fewer instances of breakage.

## Conclusion:

Knotless sutures have garnered immense popularity due to their manifold advantages in modern maxillofacial surgery. However particular constraints are also associated with knotless barbed sutures. Specifically, they cannot be employed for simple interrupted suturing technique, as it necessitates multiple points of tissue anchorage to retain the sutures. Furthermore, if the surgical site necessitates revisiting for any specific clinical indication, the suture's firm engagement with the tissues can make its removal a traumatic experience. Owing to the small sample size of current study, additional studies with large number of patients are still required to assess the effectiveness of knotless barbed suture particularly when used intraorally in the posterior region. To conclude, maxillofacial surgeons must possess a comprehensive understanding of wound healing and suture material biomechanics in order to select the most suitable materials for closure of wound.

## Figures and Tables

**Table 1:** Patient allocation and grouping

**Group**	**S. Nos. of the Patients**
GROUP I	1, 4, 5, 9, 11, 12, 13, 16, 17, 19, 22, 25, 26, 27, 28:
(study)knotless	Total = 15
GROUP II	2, 3, 6, 7, 8, 10, 14, 15, 18, 20, 21, 23, 24, 29, 30:
(control) vicryl	Total = 15

**Table 2:** Wound evaluation criteria

**Parameter**	**Yes (0)**	**No (1)**
Step off borders (when edges were not in same plane)	-	-
Contour irregularities (Wrinkled skin near wound)	__-__	___-_
Wound margin separation (When gap was present between margins)	-	-
Edge inversion (Wound not properly everted)	-	-
Excessive distortion (swelling/edema/erythema/infection)	-	-
Overall cosmetic appearance (Poor/Acceptable)	Poor (0)	Acceptable (1)

**Table 3:** Demographic detail of study parameters and site distribution of fractures

	**Knotless barbed sututre (group I)**	**3-0 vicryl rapide suture (group II)**	**P value**
MEAN AGE (mean+SD) years	26.93±6.519	32.00±13.586	0.203;NS
GENDER			
No. of Males	11(73.3%)	11(73.3%)	1.000;NS
No. of Females	4(26.7%)	4(26.7%)	
PROCEDURE (ORIF)			
Mandible(n)	13(86.6%)	9(60%)	0.099;NS
ZMC (n)	2(13.3%)	6(40%)	
NS ;p> 0.05;Non significant
n = number of fracture sites
ORIF open reduction and internal fixation

**Table 4:** Comparative evaluation of mean time taken for wound closure and length of suture material used in two groups

**Variables**	**Mean ± Standard Error**		**Mean Difference**	**t-Value#**	**p-value (Unpaired t-test)**
	Group-I (n=15)	Group-II (n=15)			
Time (min)	3.809±0.253	6.923 ± 0.438	-3.114	5.952***	< 0.001
Suture Length (cm)	15.867± 0.617	23.667 ± 1.253	-7.8	5.394***	< 0.001
# At 28 degrees of freedom
n = no of fracture sites
*** Significant at 0.1% probability level
p≥ 0.05 non-significant
p≤0.05 (5%) significant (*)
p≤0.01 (1%) very significant (**)
p≤0.001 (0.1%) highly significant (***)

**Table 5:** Mean scores of postoperative pain in two groups at different time intervals

**Time (post op)**	**Mean ± Standard Error**		**Mean Difference**	**Mann Whitney U Statistic**	**p-value**
	Group-I (n=15)	Group-II (n=15)			
Day-03	3.667 ± 0.385	5.133 ± 0.470	-1.466	54.0*	0.0144
Day-07	2.467 ± 0.264	3.733 ± 0.485	-1.266	61.5*	0.0331
Day-14	1.333 ± 0.243	2.600 ± 0.363	-1.267	54.5*	0.0143
Day-21	0.200 ± 0.667	0.667 ± 0.243	-0.467	82.0NS	0.109
n = no of fracture sites
Significant at 5% probability level;
NS Non-significant
p≥ 0.05 non-significant
p≤0.05 (5%) significant
p≤0.01 (1%) very significant
p≤0.001 (0.1%) highly significant

**Table 6:** Two-way contingency table showing different levels of wound healing in two Groups - 3rd, 7th, 14th and 21st postoperative day

**Wound Healing Score**	**Groups**		**Total**
	Group-I(n)	Group-II(n)	
3rd Postoperative Day			
3	2	0	2
4	1	8	9
5	10	7	17
6	2	0	2
Total	15	15	30
P-value for Fisher's exact test = 0.00014; (at 0.1% level of significance).			
7TH Postoperative Day			
4	2	4	6
5	3	11	14
6	10	0	10
Total	15	15	30
P-value for Fisher's exact test = 0.00668; (at 1% level of significance).			
14th Postoperative Day			
4	1	0	1
5	2	10	12
6	12	5	17
Total	15	15	30
P-value for Fisher's exact test = 0.00778; (at 1% level of significance).			
21st Postoperative Day			
5	1	4	5
6	14	11	25
Total	15	15	30
p-value for Fisher's exact test = 0.32950;			
n = no. of fracture sites

**Table 7:** Mann-Whitney U-test to compare wound healing index between two groups (PO= Post-Operative)

**Time**	**Quantity**	**Group**		**Total**	**Mann Whitney U-Statistic**	**p-value**
		Group-I	Group-II			
3rd PO Day	Valid no.	15	15	30	149.0.	0.0932
	Mean	4.8	4.47	4.63		
	SD	0.83	0.5	0.71		
	Median	5	4	5		
	1st Quartile	5	4	4		
	3rd Quartile	5	5	5		
7th PO Day	Valid no.	15	15	30	182.5**	0.0018
	Mean	5.53	4.73	5.13		
	SD	0.72	0.44	0.72		
	Median	6	5	5		
	1st Quartile	5	4.5	5		
	3rd Quartile	6	5	6		
14th PO Day	Valid no.	15	15	30	160.0*	0.0248
	Mean	5.73	5.33	5.53		
	SD	0.57	0.47	0.56		
	Median	6	5	6		
	1st Quartile	6	5	5		
	3rd Quartile	6	6	6		
21st PO Day	Valid no.	15	15	30	135.0NS	0.1577
	Mean	5.93	5.73	5.83		
	SD	0.25	0.44	0.37		
	Median	6	6	6		
	1st Quartile	6	5.5	6		
	3rd Quartile	6	6	6		
**Significant at 1% probability level;
* Significant at 5% probability level;
• Significant at 10% probability level;
NS Non-significant

**Table 8:** Complications observed in two Groups

**COMPLICATIONS**	**Group I**		**Group II**		**Total**	
	N	%	N	%	N	%
None	13	86.7	12	80	25	83.3
Suture Extrusion	2	13.3	2	13.3	4	13.3
Total	15	100	15	100	30	100
Chi-Square Test: x^2^ = 1.040;
df = 2; p = 0.595;
Not significant
N= number of fracture sites
